# Targeting aspirin in acute disabling ischemic stroke: an individual patient data meta‐analysis of three large randomized trials

**DOI:** 10.1111/ijs.12487

**Published:** 2015-04-12

**Authors:** Douglas D. Thompson, Gordon D. Murray, Livia Candelise, Zhengming Chen, Peter A. G. Sandercock, William N. Whiteley

**Affiliations:** ^1^ Edinburgh MRC Hub for Trials Methodology ResearchCentre for Population Health SciencesUniversity of Edinburgh Medical SchoolEdinburghUK; ^2^ Neurology Unit, Ca'Granda Foundation, Maggiore Policlinico HospitalIRCCSUniversity of MilanMilanItaly; ^3^ Clinical Trial Service UnitUniversity of OxfordOxfordUK; ^4^ Centre for Clinical Brain SciencesWestern General HospitalUniversity of EdinburghEdinburghUK

**Keywords:** individual patient data meta‐analysis, prediction, randomized clinical trials, stratified treatment, stroke

## Abstract

**Background:**

Aspirin is of moderate overall benefit for patients with acute disabling ischemic stroke. It is unclear whether functional outcome could be improved after stroke by targeting aspirin to patients with a high risk of recurrent thrombosis or a low risk of haemorrhage.

**Aims:**

We aimed to determine whether patients at higher risk of thrombotic events or poor functional outcome, or lower risk of major haemorrhage had a greater absolute risk reduction of poor functional outcome with aspirin than the average patient.

**Methods:**

We used data on individual ischemic stroke patients from three large trials of aspirin vs. placebo in acute ischemic stroke: the first International Stroke Trial (*n* = 18 372), the Chinese Acute Stroke Trial (*n* = 20 172) and the Multicentre Acute Stroke Trial (*n* = 622). We developed and evaluated clinical prediction models for the following: early thrombotic events (myocardial infarction, ischemic stroke, deep vein thrombosis and pulmonary embolism); early haemorrhagic events (significant intracranial haemorrhage, major extracranial haemorrhage, or haemorrhagic transformation of an infarct); and late poor functional outcome. We calculated the absolute risk reduction of poor functional outcome (death or dependence) at final follow‐up in: quartiles of early thrombotic risk; quartiles of early haemorrhagic risk; and deciles of poor functional outcome risk.

**Results:**

Ischemic stroke patients who were older, had lower blood pressure, computerized tomography evidence of infarct or more severe deficits due to stroke had increased risk of thrombotic and haemorrhagic events and poor functional outcome. Prediction models built with all baseline variables (including onset to treatment time) discriminated weakly between patients with and without recurrent thrombotic events (area under the receiver operating characteristic curve 0·56, 95% CI:0·53–0·59) and haemorrhagic events (0·57, 0·52–0·64), though well between patients with and without poor functional outcome (0·77, 0·76–0·78) in the International Stroke Trial. We found no evidence that the net benefit of aspirin increased with increasing risk of thrombosis, haemorrhage or poor functional outcome in all three trials.

**Conclusions:**

Using simple clinical variables to target aspirin to patients after acute disabling stroke by risk of thrombosis, haemorrhage or poor functional outcome does not lead to greater net clinical benefit. We suggest future risk stratification schemes include new risk factors for thrombosis and intracranial haemorrhage.

## Background

For the majority of ischemic stroke patients who are not currently eligible for intravenous thrombolysis, more effective approaches are needed to further reduce the risk of poor functional outcome. Early aspirin is an established treatment for disabling ischemic stroke, but is only modestly effective in reducing death or dependence. For the average stroke patient, it reduces the risk of poor functional outcome (death or dependence) a few months after the stroke by about 1% 1. The net clinical benefit with early aspirin treatment is probably as a result of a favorable balance of a greater reduction of thrombotic events than an increase in extra‐ and intra‐cranial haemorrhage. Therefore, the balance between the risk of haemorrhagic and thrombotic events with antiplatelets is an attractive target for a better treatment strategy for patients with acute disabling stroke.

More intensive antiplatelet regimes have not proved more effective than aspirin in acute disabling stroke. For example, GpIIb/IIIa inhibitors do not lead to an improvement in functional outcome compared to aspirin alone, perhaps because they increase the risk of intracranial haemorrhage. However, when clopidogrel and aspirin were targeted to Chinese patients with a particularly high risk of recurrent ischemic stroke, and low risk of haemorrhage – patients with recent TIA – the more intensive regime led to a net clinical benefit: fewer ischemic strokes, with no increase in intracranial haemorrhage 2. This hypothesis is of great interest in recent TIA, and being explored in different populations (Platelet Orientated Inhibition in New TIA, POINT, NCT00991029) and with more intensive antiplatelet regimes (Triple Antiplatelets for Reducing Dependence After Ischaemic Stroke, TARDIS, ISRCTN47823388).

We aimed to test the hypothesis that targeting of early antiplatelet agent on patients with a higher predicted risk of thrombosis, a lower predicted risk of haemorrhage, or a higher predicted risk of poor functional outcome leads to a lower risk of poor functional outcome by performing an individual patient data meta‐analysis.

## Methods

### Trial data

We identified trials of aspirin vs. placebo or control in acute ischemic stroke using the latest Cochrane review of antiplatelet for acute ischemic stroke 1. We excluded two small trials (one unpublished) which recruited a total of 521 patients between them, as we were unable to obtain individual patient data 3, 4. We used individual patient data from the largest randomized trials of aspirin vs. control in acute ischemic stroke patients: the International Stroke Trial (IST), the Chinese Acute Stroke Trial (CAST) and the Multicentre Acute Stroke Trial (MAST) 5, 6, 7. Although old, these three trials have the overwhelming majority of the randomized evidence on aspirin vs. placebo in acute ischemic stroke. We excluded patients where the stroke at randomization was due to intracranial haemorrhage.

### Definition of baseline characteristics and outcomes

We used baseline characteristics common to all trials with the definitions from each trial. We measured impairment due to stroke as: a weakness of arm or leg; the presence of aphasia; hemianopia; visuospatial disorder; and brainstem or cerebellar deficits. The remaining variables were: patient age, systolic blood pressure (SBP), presence of atrial fibrillation, delay from stroke to randomization in hours, and evidence of infarct on computerized tomography (CT).

We defined two early outcome events: ‘thrombotic events’ [deep vein thrombosis (DVT), pulmonary embolus (PE), ischemic stroke, and myocardial infarction (MI)] and ‘haemorrhagic events’ (significant intracranial haemorrhage, major extracranial haemorrhage, or haemorrhagic transformation of an infarct) as close as possible to 14 days after stroke. In IST events in hospital were recorded at 14 days, in MAST up to 10 days, and in CAST we restricted analysis to 14 days post randomization. We defined ‘poor functional outcome’ at final follow‐up as: a modified Rankin scale score of 3–6 in MAST; being either dead or responding ‘yes’ to the question ‘did you need help from another person to perform everyday activities within the last two weeks?’ in IST; or as dead or dependent at discharge in CAST. Additionally, we defined an ordinal outcome scale which was common across each trial. Both IST and CAST used a similar measurement of functional recovery at the end of follow‐up; we mapped the mRS used in MAST onto this common scale (see Table S1).

IST and MAST measured functional outcome at six‐months and CAST at 28 days. We made no specific allowance for differing follow‐up times as we made within‐study comparisons of the proportion of patients with poor functional outcome at the end of follow‐up.

### Model development: predicting benefit and harm

We defined baseline risk of short term adverse outcomes with clinical prediction models.

We used a non‐random split of the IST trial data to develop and internally evaluate new clinical prediction models since a random split would yield overly optimistic measures of performance 8. We developed models in patients recruited to UK and Italian centers and evaluated them in the patients recruited in the remaining 34 countries. We developed three multivariable binary logistic regression models: the first to predict thrombosis within 14 days in patients allocated to control; the second to predict haemorrhage in those allocated to aspirin; and the third to predict poor functional outcome at final follow‐up in those allocated to control. We did not try to reduce the number of variables using data dependent methods as few clinical variables were common to all trials and all of these were plausibly associated with each outcome. We assessed linearity and additivity using: restricted cubic splines (knots 3, 4 and 5) and two‐way interactions. We measured an improvement in model fit when assessing linearity and additivity as an increase in the Akaike's Information Criterion (AIC) on the chi‐squared scale which simultaneously penalizes for the added complexity 9, 10. We imputed missing data to generate multiple datasets (m = 20) since a complete case analyses can result in a reduction of statistical power and biased estimates 10.

### Model evaluation

We calculated two measures of model performance: ‘calibration’ and ‘discrimination’ 10. Calibration summarizes how well the observed events match the predicted events by dividing the cohort into groups of predicted risk and comparing the mean predicted risk with the observed frequency. We calculated the calibration slope and intercept as measures of calibration (where slope 1 and intercept 0 indicates a perfectly calibrated model). Discrimination summarizes how well a model separates patients with the event from those without. We calculated the area under the receiver operating characteristic curve (AUROCC) which ranges from no better than chance (0·5) to perfect (1·0) as a measure of discrimination. We pooled measures of performance within the evaluation split across the 20 multiply imputed sets 11.

### Estimating treatment effects in different risk groups

We defined risk groups of patients by quarters of predicted thrombotic risk and quarters of predicted haemorrhagic risk. New patient populations often differ from the model derivation data; as a consequence predicted risks may be inaccurate. To account for this, we adopted a simple updating procedure adjusting for any observed differences in outcome incidence between trials 12. In each of the risk groups we pooled estimates of the absolute risk reduction of poor functional outcome across the three trials using the Mantel–Haenszel meta‐analytical method. We examined the effect of aspirin in ten groups of patients defined by tenths of predicted risk of poor functional outcome and calculated the observed absolute risk reduction of poor functional outcome within each stratum. We contrast this empirical effect with the theoretical effect obtained under the assumption that there is no interaction between treatment with aspirin and the control event rate. Here the individualized reduction in risk equals the absolute predicted risk for the patient on control multiplied by the relative constant treatment effect from aspirin 13. Finally, because a dichotomized outcome risks losing power, we calculated the relative effect of aspirin on functional outcome measured on a common ordinal scale with ordinal logistic regression, and looked for a difference in treatment effect by continuous predicted risk on the log odds scale with multiplicative interaction terms between aspirin and the predicted risks of haemorrhage, thrombosis and poor functional outcome. We performed a sensitivity analysis excluding those patients randomized to streptokinase or high dose heparin, as these agents may increase the risk of haemorrhage in combination with aspirin and are not widely used in acute ischaemic stroke. We additionally excluded venous thrombotic events from our definition of an early thrombotic event to assess what impact this had on our results.

We used R version 3.0.1 for statistical analysis. Design and conduct of the study; collection, management, analysis, and interpretation of the data; preparation, review, or approval of the manuscript; and decision to submit the manuscript for publication was conducted independently from the study funders.

## Results

We obtained individual patient data from three trials of aspirin vs. control on a total of 39 166 patients with acute ischemic stroke. The design features of each trial, baseline patient characteristics and outcomes in follow‐up are summarized online (see Tables S2–S4). The proportion of patients with outcome events differed by trial: in IST 6·1% of patients had a thrombotic event and 1·4% had a haemorrhagic event; the corresponding proportions in CAST were 1·9% and 1·0% and in MAST 1·6% and 5·8%. About two‐thirds of patients in IST (62·1%) and MAST (63·7%) had a poor functional outcome by six‐months, and about a third of patients in CAST (30·8%) had poor functional outcome by 28 days. With aspirin, the absolute reduction in thrombotic events across trials was 6 per 1000 (95% CI 3 to 10, *P* = 0·0004, P_het_ = 0·0123), the absolute increase in haemorrhagic events across trials was 5 per 1000 (95% CI: 3 to 7, *P* < 0·0001, P_het_ = 0·4666) and the absolute reduction in poor functional outcome by six‐months across trials was 12 per 1000 (95% CI 2 to 21, *P* = 0·0135, P_het_ = 0·9193). See Fig. S1 for the associated forest plots. A similar proportion of patients had a poor functional outcome after a haemorrhagic event (446/550, 88%) and a thrombotic event (1353/1534, 88%) by last follow‐up. Median time to death was the same in patients with a thrombotic event (seven‐days, IQR 3 to 12) and with haemorrhagic event (seven‐days, IQR 3 to 11).

### Prediction of 14‐day events after acute ischemic stroke

We used multivariable logistic regression to model 14‐day thrombosis using all available baseline variables (Table 1). Only increasing age (OR: 1·21 per decade, 95% CI: 1·07 to 1·38) and the presence of a CT visible infarct (OR 1·52, 95% CI: 1·17 to 1·98) were associated significantly with increased risk of thrombotic events in the model. The final model using all baseline variables discriminated poorly between patients with and without 14‐day thrombosis (AUROCC 0·56, 95% CI 0·53 to 0·59) and was poorly calibrated (calibration slope 0·46, 95% CI: 0·33 to 0·60) (Table 2) in the evaluation split. There was no evidence of deviations from linearity.

**Table 1:** Multivariable prediction models for 14‐day events and six‐month death or dependency in the development split with imputed IST data (over 20 imputed sets)

Variable	14‐day events				Six‐month outcome	
	Thrombosis (291/4504)		Haemorrhage (74/4511)		Dead or dependent (3225/4504)	
	OR (95% CI)	*P*‐value	OR (95% CI)	*P*‐value	OR (95% CI)	*P*‐value
Age (per 10 years)						
Age	1·21 (1·07 to 1·38)	0·0027	1·24 (0·97 to 1·58)	0·086	0·44 (0·25 to 0·79)	0·0063
Age^2^	–	–	–	–	1·10 (1·05 to 1·15)	< 0·0001
SBP (per 10 mmHg)						
SBP	0·98 (0·93 to 1·02)	0·30	0·98 (0·90 to 1·08)	0·74	0·69 (0·53 to 0·91)	0·0083
SBP^2^	–	–	–	–	1·01 (1·00 to 1·02)	0·0058
Delay from randomization (hours)	0·99 (0·98 to 1·00)	0·064	1·00 (0·98 to 1·01)	0·64	1·00 (1·00 to 1·01)	0·20
Gender (Male)	1·22 (0·95 to 1·57)	0·11	1·46 (0·90 to 2·37)	0·12	0·72 (0·62 to 0·84)	< 0·0001
Visible infarct on CT	1·52 (1·17 to 1·98)	0·0019	1·12 (0·66 to 1·90)	0·68	1·05 (0·89 to 1·25)	0·56
Conscious (Drowsy/Coma vs. Alert)	0·97 (0·71 to 1·34)	0·86	1·00 (0·54 to 1·86)	0·99	4·48 (3·36 to 5·98)	< 0·0001
Presence of atrial fibrillation	1·18 (0·88 to 1·60)	0·27	1·24 (0·67 to 2·30)	0·49	1·11 (0·89 to 1·38)	0·33
At least one or more of arm/leg deficits	1·54 (0·99 to 2·39)	0·056	1·36 (0·56 to 3·28)	0·49	2·58 (2·08 to 3·19)	< 0·0001
Presence of aphasia	1·03 (0·79 to 1·33)	0·84	0·70 (0·42 to 1·15)	0·16	1·44 (1·24 to 1·69)	< 0·0001
Presence of hemianopia	1·17 (0·81 to 1·69)	0·39	1·40 (0·68 to 2·86)	0·36	2·06 (1·59 to 2·67)	< 0·0001
Presence of visuospatial disorder	1·11 (0·77 to 1·60)	0·58	1·04 (0·52 to 2·06)	0·92	1·83 (1·41 to 2·38)	< 0·0001
Presence of brainstem/cerebellar deficit	1·41 (0·95 to 2·09)	0·092	0·71 (0·28 to 1·84)	0·48	0·83 (0·66 to 1·06)	0·15

**Table 2:** Performance of prediction models in evaluation split with imputed IST data (over 20 imputed sets) for prediction models

Measure	Thrombosis (on control)	Haemorrhage (on aspirin)	Dead or dependent (on control)
Events/Total	337/4685	87/4672	2587/4685
*r* ^2^ (%) (IQR)	0·58 (0·55 to 0·59)	0·57 (0·39 to 0·67)	28·58 (28·46 to 28·82)
AUROCC	0·56 (0·53 to 0·59)	0·57 (0·52 to 0·64)	0·77 (0·76 to 0·78)
Calibration			
Intercept	0·14 (0·09 to 0·20)	0·20 (0·094 to 0·31)	−0·75 (−0·78 to −0·71)
Slope	0·46 (0·33 to 0·60)	0·52 (0·26 to 0·78)	0·90 (0·87 to 0·93)

Cell entries are estimates and 95% CI unless otherwise stated.

A multivariable logistic regression for 14‐day haemorrhage (Tables 1 and 2) discriminated poorly between those with and those without 14‐day haemorrhage (AUROCC 0·57 with 95% CI 0·52 to 0·64) and was poorly calibrated (calibration slope 0·52, 95% CI: 0·26 to 0·78) in the evaluation split. Although no one factor was significantly associated with haemorrhage at 14 days in the final model, the size and direction of all associations were similar to those for thrombotic events. There was no evidence of deviation from linearity.

### Prediction of six‐month poor functional outcome after acute ischemic stroke

A multivariable binary logistic regression to predict six‐month poor functional outcome discriminated well (AUROCC 0·77, 95% CI 0·76 to 0·78) and had good calibration (calibration slope 0·90 and intercept −0·75) in internal evaluation (Tables 1 and 2). The model had similar discrimination in the CAST (0·71, 95% CI: 0·70 to 0·72) and MAST trials (0·76, 95% CI: 0·70 to 0·81). Markers of increasing impairment due to stroke (arm/leg weakness, aphasia, hemianopia, visuospatial disorder and unconscious or drowsy at randomization) were associated with a statistically significant increase in the risk of poor functional outcome. The inclusion of a squared term for both age [Likelihood Ratio (LR) test with a *P*‐value < 0·0001] and blood pressure (LR test *P*‐value = 0·0004) resulted in a significant improvement in the prediction of poor functional outcome.

### Absolute benefit or harm of aspirin across strata of predicted risk

**Figure 1:**
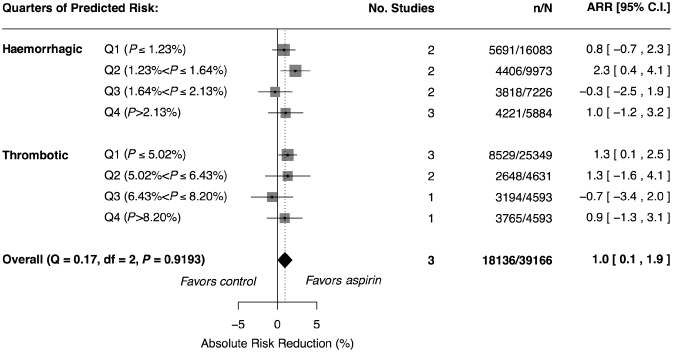
Pooled estimates of absolute risk reduction (ARR) in poor outcome. Q1 to Q4 denote quarters of risk defined on IST data. Square sizes are proportional to the strata specific denominator. Data from IST contributed to all within strata estimates, CAST contributed to most and MAST contributed to only one. Note that: No. Studies, number of studies; *n*, number of outcomes; and N, total number of patients.

**Figure 2:**
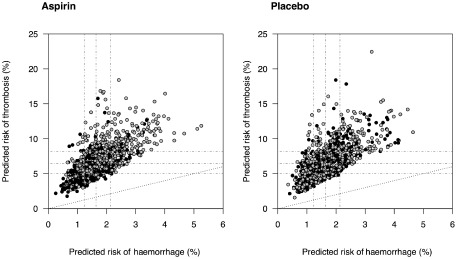
Predicted risk of thrombosis vs. predicted risk of haemorrhage in IST. Horizontal and vertical grey lines indicate quarters of risk. Grey points indicate patient dead or dependent and black alive and independent. To aid visualisation a simple random sample of 2000 patients is shown.

We defined strata of patients by quarters of predicted risk of thrombotic and haemorrhagic events on updated predictions (see Table S5). In no group was there a statistically significant increase in the risk of poor outcome with aspirin treatment compared to control. No trend of harm or benefit with aspirin was evident with increasing thrombotic or haemorrhagic risk (see Fig. S2 and Fig. 1). We plotted the risk of thrombotic events vs. the risk of haemorrhagic events in control and aspirin arms for IST (see Fig. S3 and Fig. 2). This figure demonstrates the correlation between haemorrhagic and thrombotic risk. There was no evidence that those with a high haemorrhagic risk and a low thrombotic risk were harmed by aspirin, nor was there evidence that those with a low haemorrhagic risk and high thrombotic risk benefitted any more than the average estimate of 1% reduction in poor functional outcome (though there were very few patients in these groups). In no risk group of haemorrhage or thrombotic events was the absolute reduction in poor functional outcome at final follow‐up statistically different from the overall estimate of 1% (95% CI: 0% to 2%, *P* = 0·0135, P_het_ = 0·9193 between groups). In a sensitivity analysis, we excluded patients from MAST randomized to streptokinase and from IST randomized to high dose heparin. This made no major difference to these results. Excluding venous thrombotic events from our analysis made no qualitative difference to our findings.

**Figure 3:**
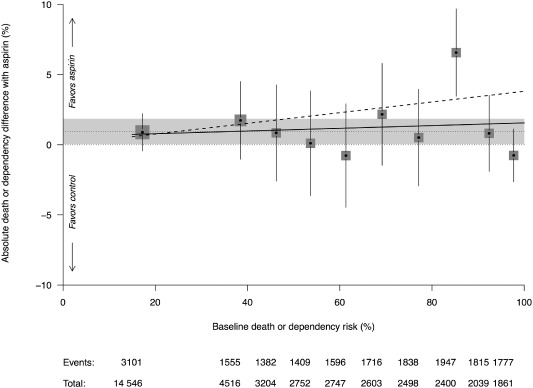
Meta‐analysis of predicted risk of poor outcome (in tenths, defined on IST data) across all three trials pooled using fixed‐effects meta‐analysis. Square sizes are proportional to the strata specific denominator. Fitted line had a slope (solid line) that was no different from zero (*P* = 0·6562) and no different from the theoretical slope (dashed line) of 0·06 (*P* = 0·1588). The dotted line and grey band represents the overall ARR estimate. In all but the seventh decile was there significant between trial heterogeneity (*P* = 0·0245).

In tenths of increasing predicted risk of poor functional outcome, there was no suggestion that patients with higher predicted risk of poor functional outcome had a greater or lesser benefit from aspirin than the overall estimate of 1% (estimated slope through pooled estimates was 0·01 with 95% CI −0·05 to 0·08, Fig. 3). Models which assumed a fixed absolute risk reduction of 1% or a constant OR of 1·06 with treatment fitted the data equally well. Using a more statistically efficient ordinal analysis modeling risk continuously, there was no evidence that the predicted risk of thrombosis and haemorrhage (*P* = 0·2244) or poor functional outcome (*P* = 0·3968) interacted with aspirin on poor functional outcome.

## Discussion

We developed and validated a reliable method to predict poor functional outcome in patients randomized to the three largest trials of aspirin therapy in acute ischemic stroke. However, there was no evidence that patients with higher predicted risks of poor functional outcome had a different response to early aspirin treatment than patients at a lower risk either on an absolute or relative scale. As our models for thrombotic and haemorrhagic events performed poorly, we were unable to identify reliably those patients at a lower risk of post‐stroke haemorrhage or higher risk of recurrent thrombotic events, who might be expected to benefit more than average from treatment with early aspirin. Using the methods we developed, there was no evidence that patients at higher predicted risk of either haemorrhage or thrombosis responded differently to aspirin than patients at a lower risk.

Our study had a number of methodological strengths: we analyzed almost all the patients randomized to early aspirin vs. control in acute stroke trials; we modeled harms and benefits separately; we used an outcome measuring disability, rather than just events, so avoiding problems assigning weight to events of different severities; we did not implicitly assume linearity or additivity in our clinical prediction models; we accounted for missing data using multiple imputation avoiding the potential biases of a complete case analysis; we left continuous predictors continuous; and finally, we did not remove insignificant predictors in our models or screen predictors at a univariate level, which would risk the problems with data‐dependent selection 14. A previous meta‐analysis did not consider the risk and benefits of treatment separately, nor data from the MAST trial 15. While a similar analysis in routinely collected data would add more patients and therefore yield greater statistical power the conclusions from such a study would be limited by the selection biases associated with the decision to treat or not treat individual patients.

Our study had a number of limitations. We used data from pragmatic trials, where the primary aim was detection of functional outcome at final follow‐up, therefore the trials probably under ascertained thrombotic and haemorrhagic events; to mitigate this limitation we developed and tested models for the thrombotic and haemorrhagic outcome in the IST trial, where the number of reported events was consistent with contemporary literature reports of post stroke complications 16. In addition, these outcomes were not adjudicated in IST or CAST. It is plausible that relative effects of early aspirin on the risk of haemorrhage are different in patients randomized to heparin or streptokinase compared to patients not on these medications. We therefore excluded, in a secondary analysis, participants randomized to these treatments which made no difference to the magnitude or direction of our results. However, as this secondary analysis did not examine patients in their randomized groups, the causal inferences that can be drawn from it are limited. We had limited sensitivity for the effect of stroke severity on thrombotic or haemorrhagic risk, as the trials measured impairment by counting relatively crude deficits, rather than using a more sensitive scale such as the National Institutes of Health Stroke Scale. We were unable to test other plausible variables that might discriminate between thrombotic or haemorrhagic events, because they were not recorded in these very large pragmatic trials, for example: previous gastrointestinal ulceration, prior DVT, cancer, cerebral microbleeds, more advanced brain imaging findings or physiological or genetic markers of aspirin metabolism. Better prediction models constructed using these variables might better identify patients who are at a higher risk of bleeding, but they would need to be implemented in new trials of antiplatelet agent in acute stroke in order to determine whether they predicted net clinical benefit or harm from treatment.

The risk of poor functional outcome at final follow‐up was the same (88%) in patients with a haemorrhagic complication to patients with a thrombotic complication. This is similar to acute coronary syndromes, where bleeding and recurrent thromboses have similar mortality (11% with recurrent MI and 12% with bleeding) 17. However, in contrast to acute coronary syndromes, where thrombosis increases mortality in the short term and haemorrhage increases mortality in the longer term, in acute stroke, short term mortality was increased by both events 18.

## Conclusions

Contrary to expectations, we found no evidence to support a stratified approach to early aspirin treatment in acute ischemic stroke patients according to the predicted risks of poor functional outcome, thrombosis or haemorrhage. Early aspirin is therefore an important part of treatment for all patients with acute disabling ischaemic stroke.

Our results do not suggest that the net clinical benefit of new acute antiplatelet regimes in acute disabling stroke will vary across subgroups defined by combinations of simple clinical variables. It is likely that such trials will need to use newer measures of the risk of thrombosis and intracranial haemorrhage in order to explore important treatment effects.

## Supporting information


**Figure S1.** Forest plots for each outcome event summarising the effect of aspirin across the three included trials.

**Figure S2.** Pooled estimates of absolute risk reduction (ARR) in poor outcome. Q1 to Q4 denote quarters of risk defined on IST data.

**Figure S3.** Predicted risk of thrombosis vs. predicted risk of hemorrhage.

**Table S1.** Defined common ordinal outcome.

**Table S2.** Characteristics of included trials.

**Table S3.** Baseline characteristics of patients randomised into controlled trials of aspirin.

**Table S4.** Outcome events in participants recruited to large randomised controlled trials of aspirin by 2 weeks after randomisation.

**Table S5.** Calibration metrics for re‐calibrated predicted risks for each trial.
