# Supplementary material for: Targeting aspirin in acute disabling ischemic stroke: an individual patient data meta‐analysis of three large randomized trials
Source: Int J Stroke. 2015 Apr 12;10(7):1024–30. doi: 10.1111/ijs.12487 (PMC4973666; doi:10.1111/ijs.12487)
Supplement: Supplementary file 1 — Figure S1. Forest plots for each outcome event summarising the effect of aspirin across the three included trials. [file IJS-10-1024-s001.doc]

| **A: Effect of aspirin on death and dependency** |
| --- |
|  |
| **B: Effect of aspirin on thrombotic events** |
|  |
| **C: Effect of aspirin on hemorrhagic events** |
|  |

**Figure S1 Forest plots for each outcome event summarising the effect of aspirin across the three included trials**
