# Supplementary material for: Targeting aspirin in acute disabling ischemic stroke: an individual patient data meta‐analysis of three large randomized trials
Source: Int J Stroke. 2015 Apr 12;10(7):1024–30. doi: 10.1111/ijs.12487 (PMC4973666; doi:10.1111/ijs.12487)
Supplement: Supplementary file 2 — Figure S2. Pooled estimates of absolute risk reduction (ARR) in poor outcome. Q1 to Q4 denote quarters of risk defined on IST data. [file IJS-10-1024-s002.doc]

Figure S2 - Pooled estimates of absolute risk reduction (ARR) in poor outcome. Q1 to Q4 denote quarters of risk defined on IST data. Square sizes are proportional to the strata specific denominator. Data from IST contributed to all within strata estimates (pooled or single), CAST contributed to most (up to the third thrombotic quarter) and MAST contributed to only one. Note that: No. Studies=number of studies; n=number of outcomes; and N=total number of patients
