# Supplementary material for: Targeting aspirin in acute disabling ischemic stroke: an individual patient data meta‐analysis of three large randomized trials
Source: Int J Stroke. 2015 Apr 12;10(7):1024–30. doi: 10.1111/ijs.12487 (PMC4973666; doi:10.1111/ijs.12487)
Supplement: Supplementary file 3 — Figure S3. Predicted risk of thrombosis vs. predicted risk of hemorrhage. [file IJS-10-1024-s003.doc]

Figure S3 Predicted risk of thrombosis vs. predicted risk of hemorrhage. Horizontal and vertical grey lines indicate quarters of risk. Grey points indicate patient dead or dependent and black alive and independent. To aid visualisation a random sample of 600 patients from each trial is shown.
