# Supplementary material for: Targeting aspirin in acute disabling ischemic stroke: an individual patient data meta‐analysis of three large randomized trials
Source: Int J Stroke. 2015 Apr 12;10(7):1024–30. doi: 10.1111/ijs.12487 (PMC4973666; doi:10.1111/ijs.12487)
Supplement: Supplementary file 4 — Table S1. Defined common ordinal outcome. [file IJS-10-1024-s004.doc]

**Table S1 Defined common ordinal outcome**

|  | **Ordinal category level (1 to 4)** | | | |
| --- | --- | --- | --- | --- |
| **Trial** | **1** | **2** | **3** | **4** |
| IST-1 | Dead | Dependent | Not recovered | Recovered |
| CAST | Dead | Partly dependent OR severely disabled | Partly recovered, but independent | Fully recovered |
| MAST-I | mRS = 6 | mRS = 3 to 5 | mRS = 2 | mRS = 0 to 1 |

Abbreviations: modified Rankin Scale - mRS
