# Supplementary material for: Targeting aspirin in acute disabling ischemic stroke: an individual patient data meta‐analysis of three large randomized trials
Source: Int J Stroke. 2015 Apr 12;10(7):1024–30. doi: 10.1111/ijs.12487 (PMC4973666; doi:10.1111/ijs.12487)
Supplement: Supplementary file 5 — Table S2. Characteristics of included trials. [file IJS-10-1024-s005.doc]

Table S2 Characteristics of included trials

|  | **IST** | **CAST** | **MAST** |
| --- | --- | --- | --- |
| Aspirin dose | 300mg | 160 mg | 300mg |
| Duration of randomised treatment | 14 days | 28 days | 10 days |
| Comparator | Avoid aspirin | Placebo | Avoid aspirin |
| Other treatments in factorial design | Subcutaneous heparin 5000 IU vs subcutaneous heparin 12 500 IU vs avoid heparin |  | Intravenous infusion of 1.5 MU streptokinase vs avoid streptokinase |
| Randomised | Factorial | 1:1 | Factorial |
| Recruitment start-end MM/YYYY | 01/1991-05/1996 | 11/1993-03/1997 | 05/1991-02/1995 |
| Definition of dead or dependent | IST score | CAST score | Modified Rankin score |
| Date of follow up for death or dependence | Six months | 28 days | Six months |

**Abbreviations:** IU = International units, MU = Million units IST = the first International Stroke Trial; CAST = the Chinese Acute Stroke Trial; MAST = the Italian Multi-centre Acute Stroke Trial etc.
