# Supplementary material for: Targeting aspirin in acute disabling ischemic stroke: an individual patient data meta‐analysis of three large randomized trials
Source: Int J Stroke. 2015 Apr 12;10(7):1024–30. doi: 10.1111/ijs.12487 (PMC4973666; doi:10.1111/ijs.12487)
Supplement: Supplementary file 6 — Table S3. Baseline characteristics of patients randomised into controlled trials of aspirin. [file IJS-10-1024-s006.doc]

Table S3 Baseline characteristics of patients randomised into controlled trials of aspirin

|  | **IST** |  | **CAST** |  | **MAST** |  |
| --- | --- | --- | --- | --- | --- | --- |
|  | No. | % | No. | % | No. | % |
|  | N = 18372 |  | N = 20172 |  | N = 622 |  |
| **Age (per year)** | | | | | | |
| Median (IQR) | 73 (65 to 80) | - | 64 (57 to 70) | - | 71 (62 to 78) | - |
| Missing | - | - | 66 | <1 | - | - |
| **Sex** |  |  |  |  |  |  |
| Women | 8517 | 46 | 7413 | 37 | 287 | 46 |
| Men | 9855 | 54 | 12759 | 63 | 335 | 54 |
| **Stroke onset to randomisation (hours)** | | | | | | |
| Median (IQR) | 20 (9 to 29) | - | 24 (12 to 37) | - | 4 (3 to 5) | - |
| Missing | - | - | 25 | <1 | - | - |
| **Antiplatelet prior to randomisation** | | | | | | |
| No | 17630 | 96 | 18048 | 89 | - | - |
| Yes | 729 | 4 | 1590 | 8 | - | - |
| Missing | 13 | <1 | 534 | 3 | - | - |
| **OCSP stroke classification** | | | | | | |
| TACS | 4395 | 24 | - | - | - | - |
| PACS | 7395 | 40 | - | - | - | - |
| LACS | 4428 | 24 | - | - | - | - |
| POCS | 2103 | 11 | - | - | - | - |
| Missing | 52 | <1 | - | - | - | - |
| **Systolic blood pressure (mmHg)** | | | | | | |
| Median (IQR) | 160 (140 to 180) | - | 154 (140 to 180) | - | 160 (140 to 170) | - |
| Missing | - | - | 19 | <1 | 3 | <1 |
| **Presence of face deficit** | | | | | | |
| No | 4749 | 26 | 4689 | 23 | - | - |
| Yes | 13392 | 73 | 14999 | 74 | - | - |
| Not assessable[[1]](#footnote-2) | 231 | 1 | 484 | 2 | - | - |
| **Presence of arm/hand deficit** | | | | | | |
| No | 2477 | 13 | 2053 | 10 | - | - |
| Yes | 15781 | 86 | 17958 | 89 | - | - |
| Not assessable | 114 | 1 | 161 | 1 | - | - |
|  |  |  |  |  |  |  |
| **Presence of leg/foot deficit** | | | | | | |
| No | 4235 | 23 | 2132 | 11 | - | - |
| Yes | 13899 | 76 | 17872 | 89 | - | - |
| Not assessable | 238 | 1 | 168 | 1 | - | - |
| **Presence of either arm/leg deficit[[2]](#footnote-3)** | | | | | | |
| No | 2194 | 12 | 1445 | 7 | 95 | 15 |
| Yes | 15920 | 87 | 18505 | 92 | 527 | 85 |
| Not assessable | 258 | 1 | 222 | 1 | - | - |
| **Presence of dysphasia** | | | | | | |
| No | 9777 | 53 | 9058 | 45 | 357 | 57 |
| Yes | 8041 | 44 | 10792 | 53 | 265 | 43 |
| Not assessable | 554 | 3 | 322 | 2 | - | - |
| **Presence of hemianopia** | | | | | | |
| No | 11736 | 64 | 17271 | 86 | 551 | 89 |
| Yes | 2924 | 16 | 915 | 5 | 71 | 11 |
| Not assessable | 3712 | 20 | 1986 | 10 | - | - |
| **Presence of visuospatial signs** | | | | | | |
| No | 12125 | 66 | 17453 | 87 | 540 | 87 |
| Yes | 2992 | 16 | 722 | 4 | 82 | 13 |
| Not assessable | 3255 | 18 | 1997 | 10 |  |  |
| **Presence of brainstem/cerebellar signs** | | | | | | |
| No | 14875 | 81 | 17687 | 88 | 606 | 97 |
| Yes | 2018 | 11 | 1350 | 7 | 16 | 3 |
| Not assessable | 1479 | 8 | 1135 | 6 | - | - |
| **Presence of other deficit** | | | | | | |
| No | 16064 | 87 | 17157 | 85 | - | - |
| Yes | 1143 | 6 | 1370 | 7 | - | - |
| Not assessable | 1165 | 6 | 1645 | 8 | - | - |
|  |  |  |  |  |  |  |
|  |  |  |  |  |  |  |
|  |  |  |  |  |  |  |
|  |  |  |  |  |  |  |
|  |  |  |  |  |  |  |
| **Conscious state at randomisation[[3]](#footnote-4)** | | | | | | |
| Drowsy or unconscious | 4255 | 23 | 2560 | 13 | 54 | 9 |
| Fully alert | 14117 | 77 | 17588 | 87 | 568 | 91 |
| Not assessable | - | - | 24 | <1 | - | - |
| **Atrial fibrillation** | | | | | | |
| No | 14391 | 78 | 18732 | 93 | 472 | 76 |
| Yes | 3034 | 17 | 1350 | 7 | 150 | 24 |
| Missing | 947[[4]](#footnote-5) | 5 | 90 | <1 | - | - |
| **CT evidence of infarction** | | | | | | |
| No | 12041 | 66 | 2635 | 13 | 519 | 83 |
| Yes | 6331 | 34 | 15140 | 75 | 103 | 17 |
| Missing | - | - | 2397 | 12 | - | - |

1. In IST, patients with coma, aphasia or cognitive deficits items were recorded as ‘not assessable’ if the item could not be assessed [↑](#footnote-ref-2)
2. MAST records slight/severe motor deficit present in arm/leg [↑](#footnote-ref-3)
3. Recorded in MAST censored from motor deficit [↑](#footnote-ref-4)
4. Not recorded in the 947 patients in the IST pilot study [↑](#footnote-ref-5)
