# Supplementary material for: Targeting aspirin in acute disabling ischemic stroke: an individual patient data meta‐analysis of three large randomized trials
Source: Int J Stroke. 2015 Apr 12;10(7):1024–30. doi: 10.1111/ijs.12487 (PMC4973666; doi:10.1111/ijs.12487)
Supplement: Supplementary file 7 — Table S4. Outcome events in participants recruited to large randomised controlled trials of aspirin by 2 weeks after randomisation. [file IJS-10-1024-s007.doc]

Table S4 Outcome events in participants recruited to large randomised controlled trials of aspirin by 2 weeks after randomisation. Each number is an individual with a hemorrhagic or a thrombotic event. Twenty one individuals experienced both a hemorrhagic and a thrombotic event in IST. Follow up events are reported at 14 days in IST and CAST, and at 10 days in MAST.

|  | **IST** |  | **CAST** |  | **MAST** |  |
| --- | --- | --- | --- | --- | --- | --- |
|  | No. | % | No. | % | No. | % |
|  |  |  |  |  |  |  |
| Deep venous thrombosis | 21 | 0.1 | - | - | - | - |
| Pulmonary embolism | 122 | 0.7 | 19 | 0.1 | - | - |
| Ischemic stroke | 632 | 3.4 | 319 | 1.6 | 5 | 0.8 |
| Myocardial infarction | 357 | 1.9 | 49 | 0.2 | 5 | 0.8 |
| **All thrombotic events** | **1118** | **6.1** | **380** | **1.9** | **10** | **1.6** |
|  |  |  |  |  |  |  |
| Significant intracranial hemorrhage | 119 | 0.6 | 75 | 0.4 | 32 | 5.1 |
| Major extracranial hemorrhage | 150 | 0.8 | 90 | 0.4 | 8 | 1.3 |
| Hemorrhagic transformation | 49 | 0.3 | 50 | 0.2 | - | - |
| **All hemorrhagic events** | **265** | **1.4** | **204** | **1.0** | **36** | **5.8** |
|  |  |  |  |  |  |  |
| **Dead or dependent** | **11413** | **62.1** | **6211** | **30.8** | **396** | **63.7** |
| Missing | 137 | 0.75 | 48 | 0.24 | - | - |
