# Supplementary material for: Targeting aspirin in acute disabling ischemic stroke: an individual patient data meta‐analysis of three large randomized trials
Source: Int J Stroke. 2015 Apr 12;10(7):1024–30. doi: 10.1111/ijs.12487 (PMC4973666; doi:10.1111/ijs.12487)
Supplement: Supplementary file 8 — Table S5. Calibration metrics for re‐calibrated predicted risks for each trial. [file IJS-10-1024-s008.doc]

Table S5 Calibration metrics for re-calibrated predicted risks for each trial

| **Measure** | **Thrombosis** | **Hemorrhage** | **Dead or dependent** |
| --- | --- | --- | --- |
|  | (on control) | (on aspirin) | (on control) |
|  | | | |
| IST |  |  |  |
| events/Total | 628/9189 | 161/9183 | 5812/9189 |
| Intercept | 0.00 (-0.08 to 0.08) | 0.00 (-0.15 to 0.16) | -0.08 (-0.13 to -0.04) |
| Slope | 0.68 (0.47 to 0.89) | 0.71 (0.32 to 1.09) | 0.94 (0.89 to 0.99) |
|  |  |  |  |
| CAST |  |  |  |
| events/Total | 206/10080 | 123/10092 | 3164/10080 |
| Intercept | -0.01 (-0.14 to 0.13) | 0.00 (-0.18 to 0.18) | -0.18 (-0.23 to -0.14) |
| Slope | 0.81 (0.41 to 1.21) | 0.42 (-0.08 to 0.92) | 0.92 (0.87 to 0.97) |
|  |  |  |  |
| MAST |  |  |  |
| events/Total | 4/313 | 22/309 | 203/313 |
| Intercept | -0.01 (-0.99 to 0.98) | 0.01 (-0.43 to 0.44) | 0.02 (-0.27 to 0.22) |
| Slope | 0.37 (-2.73 to 3.47) | -0.88 (-2.08 to 0.33) | 1.39 (1.00 to 1.79) |
